# A Systematic Study of the Antioxidant Capacity of Humic Substances against Peroxyl Radicals: Relation to Structure

**DOI:** 10.3390/polym13193262

**Published:** 2021-09-25

**Authors:** Olga I. Klein, Natalia A. Kulikova, Andrey I. Konstantinov, Maria V. Zykova, Irina V. Perminova

**Affiliations:** 1 Bach Institute of Biochemistry, Fundamentals of Biotechnology Federal Research Center, Russian Academy of Sciences, pr. Leninskiy 33, 119071 Moscow, Russia; klein_olga@list.ru; 2 Department of Soil Science, Lomonosov Moscow State University, Leninskiye Gory 1-12, 119991 Moscow, Russia; 3 Department of Chemistry, Lomonosov Moscow State University, Leninskiye Gory 1-3, 119991 Moscow, Russia; konstant@med.chem.msu.ru (A.I.K.); iperm@med.chem.msu.ru (I.V.P.); 4 Department of Chemistry, Siberian State Medical University, 634050 Tomsk, Russia; huminolog@mail.ru

**Keywords:** ORAC, humic acids, fulvic acids, carbohydrate, *Trametes maxiama*, ^13^C NMR solution-state spectroscopy, total phenol content

## Abstract

Humic substances (HS) are natural supramolecular systems of high- and low-molecular-weight compounds with distinct immunomodulatory and protective properties. The key beneficial biological activity of HS is their antioxidant activity. However, systematic studies of the antioxidant activity of HS against biologically relevant peroxyl radicals are still scarce. The main objective of this work was to estimate the antioxidant capacity (AOC) of a broad set of HS widely differing in structure using an oxygen radical absorption capacity (ORAC) assay. For this purpose, 25 samples of soil, peat, coal, and aquatic HS and humic-like substances were characterized using elemental analysis and quantitative ^13^C solution-state NMR. The Folin–Ciocalteu method was used to quantify total phenol (TP) content in HS. The determined AOC values varied in the range of 0.31–2.56 μmol Trolox eqv. mg^−1^, which is close to the values for ascorbic acid and vitamin E. Forward stepwise regression was used to reveal the four main factors contributing to the AOC value of HS: atomic C/N ratio, content of O-substituted methine and methoxyl groups, and TP. The results obtained clearly demonstrate the dependence of the AOC of HS on both phenolic and non-phenolic moieties in their structure, including carbohydrate fragments.

## 1. Introduction

Humic substances (HS) are ubiquitous in natural and human-made environments, such as organic rocks, soil, compost, and natural water [1,2]. Consolidated resources of humic materials are deposited mostly in peat, coal, composts, and sapropel [3]. HS are produced in situ due to chemical, physical, and microbial degradation, as well as (re)polymerization of phenolic and aromatic components such as lignin, tannins, polysaccharides, lipids, and proteins [4,5,6]. They have a non-stoichiometric elemental composition, irregular structure, and heterogeneous molecular composition [7]. A single structural formula cannot be assigned to any sample of HS. As a result, HS are still operationally classified according to their solubility in acidic and alkaline solutions into two major classes: humic acids (HA), which are insoluble at pH < 2, and fulvic acids (FA), which are soluble in the whole pH range [8].

Recently, HS have attracted increasing attention from the point of view of their use in medicine [9] due to their therapeutic potential against chronic inflammatory diseases [10,11,12], antiviral activity [13,14,15], beneficial effect in accelerating cutaneous wound healing [16,17], and chelating activity toward toxic metals [18,19].

Antioxidant properties of HS against reactive oxygen species (ROS) and free radicals are generally believed to largely determine their significant potential for use in medicinal, pharmaceutical, and cosmetic application and food industries [9,19,20,21,22]. Piotrowska and co-workers reported suppression of lipid peroxidation in human placenta mitochondria in the presence of peat HS, confirmed by a decreased malondialdehyde level [23]. The reported hepatoprotective [24,25,26], neuroprotective [20], renoprotective [27], and cardioprotective [28,29] effects of HS were explained by their antioxidant activity.

ROS or high levels of free radicals cause oxidative stress, leading to degradation of DNA, cell membranes, proteins, and other cellular constituents [30,31,32]. This may induce a number of human diseases and conditions, such as atherosclerosis, rheumatoid arthritis, muscular dystrophy, cataracts, neurological disorders, some types of cancers, and aging [33]. Oxidative stress can be ameliorated by supplying effective antioxidants [34,35]. Epidemiological studies of dietary antioxidant consumption and health outcomes have demonstrated the protective effects of various antioxidant-rich foods against chronic diseases (cardiovascular disease, neurodegeneration, and cancer), myocardial infarction, and stroke. Regular dietary antioxidant intake results in increased total antioxidant capacity in the serum and decreased plasma levels of inflammatory cytokines (IL-6 and TNF-α), the oxidative stress biomarker F2-isoprostane, and a nonspecific marker of inflammation in serum, C-reactive protein (CRP) [36]. The antioxidant activity of HS has been attributed mainly to the presence of phenolic and quinoid moieties. Similar to phenols, HS can behave as electron donors or acceptors depending on the redox state of the system [21,37]. This paradigm is in good agreement with the observation that phenol moieties are structural fragments responsible for the antioxidant activity of many other naturally occurring bioactive substances [9,38,39]. Still, very little is known about the non-phenolic moieties of HS, which could also contribute to their antioxidant properties. Non-phenolic antioxidants represent an important and abundant class of natural radical scavengers [34]. Over the past decade, a variety of natural polysaccharides have attracted great interest due to their antioxidant functions, responsible for wide-ranging beneficial therapeutic effects and health-promoting properties [40,41]. The content of carbohydrates in HS can reach 10% [42], which might represent their contribution to the antioxidant activity of humic materials.

More than 40 laboratory protocols for testing antioxidant capacity and their modifications are reported in the literature [43,44,45]. Among them, the oxygen radical absorption capacity (ORAC) method is relevant to in vivo conditions because it uses a biologically relevant free radical source, peroxyl radical ROO^•^, which is the most prevalent free radical in human biology [36,46]. Peroxyl radicals are characterized as free radicals that predominate in lipid oxidation in biological systems and in foodstuffs under physiological conditions [46]. The ORAC method was initially based on the ability of antioxidants to prevent the consumption of β-phycoerythrin mediated by peroxyl radicals generated during the aerobic thermal decomposition of 2,2′-azo-bis(2-amidinopropane)dihydrochloride (AAPH). However, the currently employed assay, based on work published by Ou and co-workers [47], in which fluorescein is proposed as the target molecule, is believed to determine the capacity of antioxidants to trap AAPH-derived peroxyl and/or AAPH-derived alkoxyl radicals [48]. Along with peroxyl radicals, alkoxyl radicals are also abundant, damaging free radicals in the human body [49].

The ORAC test is usually classified as a method that involves hydrogen atom transfer (HAT) reactions [36,46,48,50]. For non-phenolic antioxidant reactions, donation from other than H atoms can also occur with peroxyl radicals [34]. The presence of alkoxyl radicals complicates the situation, and they can be scavenged by reactions involving proton-coupled electron transfer (PCET), electron transfer–proton transfer (ET-PT), and second sequential proton loss electron transfer (SPLET) mechanisms [49,51]. Despite some uncertainty in our understanding of the mechanisms underlying the ORAC assay [48], this approach provides a direct measure of the hydrophilic and lipophilic chain-breaking antioxidant capacity [36] of both phenolic [50] and non-phenolic antioxidants, such as carbohydrates [52]. However, studies devoted to assessing the antioxidant activity of HS mainly use assays with the 2,2′-azinobis-(3-ethylbenzothiazoline-6-sulfonic acid (ABTS) cation radical [37,53], superoxide radical O_2_^•^ [54], reduction of the complex of ferric ions (Fe^3+^)^−^ [55], or Cu (II) reduction to Cu (I) [56], or they initiate oxidation of 1,4-dioxane as a model reaction [57]. The only study of the antioxidant activity of HS against peroxyl radical induced by α,α-azobisizobutyronytril (AIBN) was performed for a single sample of coal-derived HA [58].

The main objective of this work was to characterize HS antioxidant capacity (AOC) using the ORAC assay as a function of HS structure, which can be responsible for the type of activity of humic materials. To this end, we quantified total phenol content using the Folin–Ciocalteu method and carbon distribution among the structural fragments using quantitative ^13^C solution-state NMR of 25 samples of soil, peat, coal, and aquatic HS and humic-like substances (HLS) produced by basidiomycete fungi. To obtain insight into the antioxidant activity of HS, we developed a forward stepwise multiple linear regression. To visualize HS clustering based on physical–chemical properties responsible for antioxidant activity, we applied principal component analysis (PCA).

## 2. Materials and Methods

Ultrapure deionized water (Milli-Q^®^, 18 MΩ cm, Millipore, Burlington, MA, USA) was used to prepare all solutions. All chemicals used were of at least analytical grade. Inorganic compounds (NaOH, HCl, KCl, KH_2_PO_4_, K_2_HPO_4_) were obtained from Chimmed (Russia). Folin and Ciocalteu’s phenol reagent, 2,2′-azobis (2-amidino-propane) dihydrochloride (AAPH), fluorescein, 6-hydroxy-2,5,7,8-tetra-methylchroman-2-carboxylic acid (Trolox), hydroxybenzoic acid (HBA), vanillic acid, syringic acid, gallic acid, fumaric acid, ferulic acid, sinapic acid, ascorbic acid, and vitamin E were procured from Sigma-Aldrich (Burlington, MA, USA). Standard samples of HS, including Suwannee River humic acid (SRHA) and fulvic acid (SRFA) and dissolved organic matter (SRDOM), were kindly provided by I.V. Perminova, coordinator of the Russian chapter of the International Humic Substances Society (IHSS, St. Paul, MN, USA). Commercially available coal humic materials CHA-AGK and CHA-ALD were purchased from Biotechnology Ltd. (Moscow, Russia) and Sigma-Aldrich (Burlington, MA, USA), respectively.

### 2.1. Isolation of Humic Substances

Humic materials were isolated from natural sources including soil (9 samples), peat (9 samples), coal (2 samples), and natural water (3 samples). They were either fractionated into humic acid (HA) and fulvic acid (FA) or used as a non-fractionated mixture of HA and FA (HF). Additionally, humic-like substances (HLS) were used, synthesized during solid-phase cultivation of the basidiomycete *Trametes maxima* 0275 (2 samples). Overall, 25 samples of humic materials were used in the study. A list of the HS and their sources is presented in Table 1.

Soil HA and FA (SHA and SFA) were isolated from Albic Retisol (Moscow region, Russia) and Gleyic and Endocalcic Chernozems (Voronezh region, Russia), as described in [59]. In brief, the SHA and SFA from Albic Retisol were obtained by alkali extraction with 0.1 M NaOH. For Chernozems, before alkali extraction the sample was pretreated with 10% HCl. The alkali extract was treated with 0.3 M KCl and centrifuged to remove organomineral colloidal particles. The SHA and SFA were obtained by acidification of the supernatant to pH 1–2. The precipitated HA were desalted by dialysis. To isolate FA, the acidic supernatant was passed through XAD-2 resin. The sorbed fraction of SFA was recovered by back elution with 0.1 M NaOH, desalted on cation-exchange resin, and freeze-dried.

Peat HF (PHF) was isolated from samples of highland and lowland peat located in the Tver region (Russia). The highland peat type was sedge (T6), the lowland peat types were sedge (T3) and woody (T7). The isolation procedure was described elsewhere [60] and included preliminary treatment with an ethanol–benzene mixture (1:1) followed by alkaline extraction (0.1 M NaOH). The SHA and SFA were obtained by acidification as described for soil HA and FA.

Coal HA (CHA) was obtained from two commercial preparations, ALD (Aldrich HA) and AGK (Biotechnology Ltd., Moscow, Russia). They were desalted on a cation-exchange resin and freeze-dried.

Aquatic HS consisted of standard samples of HS produced by the International Humic Substances Society (IHSS, St. Paul, MN, USA), including Suwannee River HA (SRHA), FA (SRFA), and dissolved organic matter (SRDOM).

Two samples of HLS, HLS-45, and HLS-70 were produced in our laboratory by the basidiomycete *Trametes maxima* 0275 cultivated on oat straw. HLS-45 and HLS-70 were isolated after 45 and 70 days of solid-phase cultivation, as described previously [61]. In brief, after 45 and 70 days of cultivation, the flasks containing fungal cultures were supplemented with warm distilled water (60 °C) and left for 6 h under constant stirring. Then the liquid was filtered through a paper filter to separate the straw with mycelium. The filtrate was supplemented with concentrated HCl drop-by-drop to adjust the pH to 2.0. The solution was left for 24 h; as a result, a grayish-brown flocculent precipitate was formed. The precipitate was collected by centrifugation, washed with distilled water, centrifuged again, and dialyzed against distilled water.

### 2.2. Characterization of Humic Substances

The CHN content was determined using a model 1106 elemental analyzer (Carlo Erba Strumentazione, Milan, Italy). The oxygen content was calculated as the difference between the dry ash-free weight of the sample and the total CHN content.

Quantitative ^13^C solution-state NMR spectra were recorded on a Bruker Advance 400 spectrometer (Bruker BioSpin, Germany) operating at 100 MHz according to Hertkorn and co-authors [62]. To quantify the spectra obtained, the following assignments were made (in ppm): 220–189, C atoms of quinone and ketone groups (C_C=O_); 189–168, C atoms of carboxylic and esteric groups (C_COO_); 168–145, aromatic O-substituted C atoms (C_Ar-O_); 145–108, aromatic H- and C-substituted atoms (C_Ar_); 108–91, anomeric double substituted aliphatic C atoms (C_OCO_); 91–66, О-substituted methine groups (C_CHO_), 66–59, O-substituted methylene groups (C_CH2O_); 59–48, methoxyl C atoms (C_CH3O_), 48–0, and aliphatic H- and C substituted C atoms (C_CHn_). In addition to the integrals of the given ranges, the sum of O-substituted aliphatic C ΣC_Alk-O_ and carbohydrate carbon ΣC_Carb_ and the ΣC_Ar_/ΣC_Alk_ ratio were calculated. The value of ΣC_Alk-O_ was the sum of C_OCHO_ + C_CHO_ + C_CH2O_ + C_CH3O_, ΣC_Carb_ was the sum of C_OCO_, C_CHO_, and C_CH2O_, and ΣCAr and ΣCAlk were the sum of C_Ar_ + C_Ar–O_ and ΣC_Alk-O_ + C_CHn_, respectively.

The total phenolic (TP) content of the HS and HLS samples was determined according to the Folin–Ciocalteu method [63].

### 2.3. Estimation of Oxygen Radical Absorbance Capacity (ORAC) of Humic Substances

ORAC of HS was determined as described by Ou and co-authors [47]. For this purpose, 2,2′-azobis (2-amidino-propane) dihydrochloride (AAPH), fluorescein, and 6-hydroxy-2,5,7,8-tetra-methylchroman-2-carboxylic acid (Trolox) were prepared in 75 mM phosphate buffer with a pH of 7.4. For measurements, AAPH was prepared at a concentration of 600 mM and made fresh daily. A sodium fluorescein stock solution (4 μM) was stored wrapped in foil at 5 °C. Immediately prior to use, the stock solution was diluted with phosphate buffer to a final concentration 0.08 μM. Trolox concentration was 25 μM.

To perform the measurements, a Synergy HT Multi-Detection Microplate Reader (BioTek Instruments Inc., Winooski, VT, USA) was used, with 115 µL of working sodium fluorescein solution added to all experimental wells. Control wells received 15 µL of phosphate buffer, while standards received 15 µL of Trolox dilution and samples received 15 µL of HS solution at a concentration 10 mg L^−1^. The plate was then allowed to equilibrate by incubating for 30 min at 37 °C. Reactions were initiated by the addition of 15 µL of AAPH solution [64]. Fluorescence was then monitored kinetically with data taken every minute for 1 h. An excitation wavelength of 485 nm and an emission wavelength of 528 nm were used in the assay. The AOC value referred to the net protective area under the quenching curve of fluorescein in the presence of an antioxidant. The net area under the curve (AUC) of the standard (Trolox) and HS was calculated. The AOC related to Trolox was calculated as follows:

(1) $$\mathrm{AOC}=\frac{{\mathrm{AUC}}_{\mathrm{HS}}-{\mathrm{AUC}}_{\mathrm{control}}}{{\mathrm{AUC}}_{\mathrm{Trolox}}-{\mathrm{AUC}}_{\mathrm{control}}}\times \left[\mathrm{Trolox}\right],$$

where [Trolox] is the concentration of Trolox in micromoles.

The results were evaluated using Origin 8.0 software (OriginLab Corp., Northampton, MA, USA) and expressed as μmol of Trolox equivalent (TE) per milligram of sample. The measurements were performed in fourfold repetition. To compare antioxidant properties of HS with other antioxidants, the AOC of several individual compounds was estimated using the ORAC approach including hydroxybenzoic acid (HBA), vanillic acid, syringic acid, gallic acid, fumaric acid, ferulic acid, sinapic acid, ascorbic acid, and vitamin E.

### 2.4. Statistical Data Treatment

Data on the AOC of HS are presented as the mean ± standard deviation (SD). To analyze differences among means, analysis of variance (ANOVA) was applied, followed by Tukey’s HSD test at *p* < 0.05. To reveal the relationship between the structural characteristics and antioxidant properties of HS, a correlation analysis, principal component analysis (PCA), was applied and a forward stepwise regression model was developed. All statistical data treatments were carried out using the Statistica 8.0 software package (StatSoft, Dell Inc., Round Rock, TX, USA).

## 3. Results

### 3.1. Structural Characteristics of HS

A set of the analyzed humic materials included HS samples isolated from different natural environments and HLS produced by the basidiomycete fungus. In addition, the HS samples were of different fractional composition, including HA, FA, and non-fractionated HA and FA. This enabled substantial variation of structural characteristics within the test set (Table 2).

The data on elemental composition show that the highest amount of nitrogen was characteristic of soil humic acids and fulvic acids: the C/N ratio values varied between 11 and 20, whereas for peat HA and HF they varied from 26 to 50, reaching absolute maximum for peat FA above 100. This is indicative of the minimal content of nitrogen in peat FA. These humic materials were characterized with maximum values of the O/C ratio and the content of aliphatic units, for both carbohydrates and non-oxidized alkyl chains. They had minimum ΣC_Ar_/ΣC_Alk_ values (around 0.5), which is indicative of the highly aliphatic nature of these humic materials. The most aromatic humic materials with the lowest H/C ratio (0.6–0.8) and the highest C_Ar_ values were humic acids from Mollisol and coal.

The magnitude of the H/C ratio, which indicates the degree of aromaticity or unsaturation [65], varied from 0.62 (prevalence of aromatic fragments) to 1.22 (prevalence of aliphatic fragments). On average, aromaticity increased in the following order: HLS < freshwater HS < peat HS < soil HS < coal HS. The same trend could be observed when using the ΣC_Ar_/ΣC_Alk_ ratio as an indicator of aromaticity (the higher this ratio, the higher the aromaticity). A statistically significant correlation (r = −0.64) between H/C and ΣC_Ar_/ΣC_Alk_ was calculated (Table S1).

**Figure 1:**
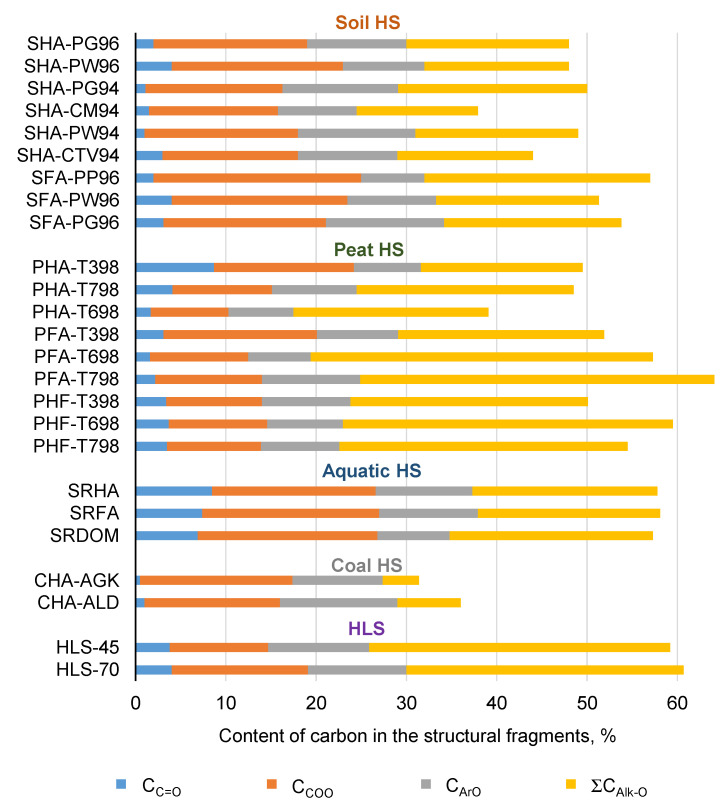
Percentage of O-containing structural fragments in humic materials used in this study as measured by ^13^C NMR spectroscopy.

The relative content of oxygen-bearing moieties, which can be indicated by the O/C ratio, or oxidation degree, was in the range of 0.31–0.66. The oxidation degree varied in the following descending order: freshwater HS > peat HS > soil HS > HLS > coal HS. Recently, polysaccharides as a source of novel potential antioxidants were reported [31,66,67,68,69,70]. Although carbohydrates do not possess strong antioxidant activity due to low numbers of aldehyde and ketone groups in their structure, a detailed study of the antioxidant activity of monosaccharides, oligosaccharides, and complex carbohydrates yielded non-zero AOC values for sugars from 0.001 to 0.022 μmol TE mg^−1^ [52]. That is why it was of interest to compare the content of oxygen-bearing fragments in HS from various sources (Figure 1).

Aquatic HS demonstrated the highest content of carboxylic groups, and coal HS had the lowest. Coal HS was characterized by its minimal content of carbohydrate fragments. In contrast, peat HS and HLS had the highest content of ΣC_Carb_. HLS showed the maximum content of methoxyl carbon, showing a high contribution of lignin fragments in the structure of these humic materials.

The obtained data on the elemental composition and carbon content of the structural fragments are in good agreement with those reported in the literature [65,71]. For instance, in a statistical evaluation of the elemental composition of HS by Rice and MacCarthy [65], the reported values for O/C and H/C of HA were 0.08–1.20 and 0.08–1.85, respectively, which coincide with the values of 0.31–0.60 and 0.62–1.10 found in our study (Table 2). For FA, O/C and H/C varied from 0.17 to 1.19 and from 0.77 to 2.13, while in our work the analogous values were 0.51–0.66 and 0.76–1.10. The distribution of carbon among major structural fragments of HS as measured by ^13^C NMR spectroscopy corroborated well with the reported data for soil HS [59,72], peat HS [73], freshwater HS [74], and coal humic materials [75].

In general, the obtained data demonstrate trends that are generally reported for HS derived from different natural resources. Coal-derived HA had the minimum O/C ratio; they were enriched with aromatic fragments ΣC_Ar_ [65,76]. HS extracted from freshwater were characterized by their maximal content of C_COO_ carboxylic groups due of high microbiological activity, resulting in oxidation of humic materials [74]. HS from peat had a high content of ΣC_Carb_ carbohydrates [73] due to low intense biological turnover in peat, resulting in incomplete microbiological decomposition of hydrocarbon residues. So, it can be concluded that the HS samples used in this study were typical representatives of humic materials isolated from the corresponding environments.

Phenolic moieties are supposed to be the most important scavengers of peroxyl radicals in different antioxidant compounds, including HS [37,38,77,78]. They convert peroxyl radicals into hydroperoxides and are themselves converted into phenoxyl radicals. A direct estimation of TP in HS demonstrated a phenol content in the range of 0.51–3.53 μmol TE mg^−1^ (Table 2). The maximum was observed for peat HA (PHA-T698) and the minimum for soil HA (SHA-CTV94). In general, peat HS were characterized by higher phenol content than other preparations. For peat HS, the TP values were in the range of 1.239–3.353 μmol TE mg^−1^, while for soil, coal, and freshwater HS, they did not exceed 1.760, 2.348, and 2.504 μmol TE mg^−1^, respectively. For the HLS samples, the TP values were 1.381–1.481 μmol TE mg^−1^, close to those observed for soil HS (0.510–1.760 μmol TE mg^−1^). As a rule, the humic acid fractions were characterized by a higher content of phenols compared to the corresponding fulvic acids. In general, the values of this parameter for HS from different sources strongly overlap.

Thus, the analysis of structural characteristics showed that the studied HS had a wide variation in parameters that might determine their antioxidant activity.

### 3.2. Antioxidant Capacity of HS

The AOC values of the HS samples isolated from different environments used in this study are shown in Table 3.

The obtained data indicate that for terrestrial sources (peat, soil), fulvic acids were characterized by much higher AOC values compared to humic acid isolated from the same source. For example, for soil humic materials, the AOC value (μmol TE mg^−1^) was 0.75 for SHA-PW96 and 1.2 for SFA-PW96; for peat HS, the corresponding values were 1.63 for PHA-T798 and 2.25 for PFA-T798. The AOC value of the non-fractionated sample of PHF-T798 was 1.56 μmol TE mg^−1^, which is very close to that of PHA-T798. This might be connected to the prevalence of humic acid fractions in the non-fractionated samples of peat HS.

Since phenolic moieties are generally believed to be responsible for the antioxidant activity of HS [39,79,80], one would expect that among the studied samples, the maximum antioxidant capacity would be observed for peat HS. Indeed, the data in Table 3 show that the highest AOC values were measured for peat HS. However, ANOVA followed by Tukey’s HSD test demonstrated that some samples of soil FA, coal HA, and freshwater HA also fell within the homogeneous groups of peat HS formed according to AOC (Table S2), indicating the proximity of their values.

AOC values of HS (0.31–2.56 μmol TE mg^−1^) were close to those of ascorbic acid (2.32 μmol TE mg^−1^) and vitamin E (2.95 μmol TE mg^−1^), but much lower than those of the other antioxidants used in the study (Table 4).

The obtained ORAC values for HS were slightly lower compared to those determined with the Trolox equivalent antioxidant capacity (TEAC) approach [53]. For HLS-45 and HLS-70, the TEAC AOC values were 3.3 and 2.9 μmol mg^−1^, respectively, which are 3–5 times larger than the corresponding ORAC AOC values (Table 3). For water HS samples, IHSS standards SRHA, SFRA, and SRDOM, the TEAC AOC values were 3.0, 2.4, and 2.5 μmol mg^−1^, respectively, which is 1.5–2.0 times larger than the ORAC values (Table 3). Of importance is that the described inconsistency between the TEAC and ORAC values for the analyzed humic materials, with the former substantially exceeding the latter, was not observed with the individual antioxidants [52,81]. Hu and co-authors reported that monosaccharides, oligosaccharides, and complex carbohydrates showed ORAC values, but not TEAC values [52]. A similar trend of lower TEAC values compared to ORAC values was reported for the phenolic antioxidants oxyresveratrol, resveratrol, pinosylvin, and pterostilbene [81]. This inconsistency might be related to the more complex antioxidant behavior of HS than individual antioxidant compounds.

**Figure 2:**
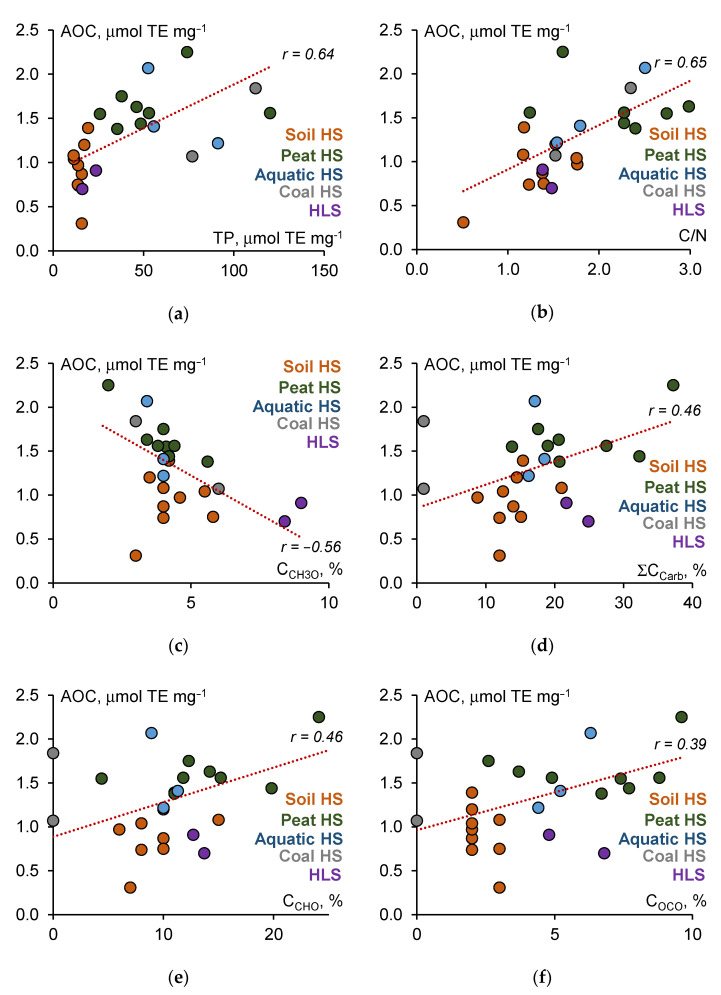
Correlation fields between AOC values of HS and their physical–chemical properties: (**a**) TP; (**b**) C/N ratio; (**c**) C_CH3O_; (**d**) ΣC_Carb_; (**e**) C_CHO_; (**f**) COCO.

Several significant correlations were found between the AOC values and structural characteristics of HS (Table S1), including TP (r = 0.64), C/N (r = 0.65), C_CH3O_ (r = −0.56), ΣC_Carb_ (r = 0.46), C_CHO_ (r = 0.46), and C_OCO_ (r = 0.39). All significant correlations between the AOC values and structural parameters of HS are shown in Figure 2.

As can be seen from the correlation dependencies presented in Figure 2, the antioxidant activity of humic materials against peroxyl radicals depended on the content of both phenolic and non-phenolic fragments in the structure of HS.

## 4. Discussion

The AOC values of HS against peroxyl radicals found in this study varied from 0.31 to 2.56 μmol TE mg^−1^. The high-end values are close to those of ascorbic acid (2.32 μmol TE mg^−1^) and vitamin E (2.95 μmol TE mg^−1^). This is in line with other studies on the antioxidant activity of HS and their comparison with other antioxidants [82,83,84]. Similar effects of ascorbic acid and HA were reported for wheat growth and attributed to their antioxidant activity [83]. Sakr and co-authors demonstrated a similar capability of ascorbic acid and HA to mitigate biotic stress in wheat plants induced by a pathogenic fungus, *Fusarium graminearum* [85]. Peat HS were shown to possess antioxidant activity close to that of vitamin E [23,84,86].

Significant correlations were found between the AOC values and structural characteristics of HS such as TP, C/N, C_CH3O_, ΣC_Carb_, C_CHO_, and C_OCO_ (Figure 2). The relationship between AOC and TP can be explained by the fact that a formal H atom donation from weak X–H bonds (where X = O, N, or S) is the major mechanism of peroxyl radical quenching. This mechanism relies on the ability of certain antioxidants (chain-breaking antioxidants) to donate H atoms to ROO^•^ [34]. This mechanism of action occurs for phenols and antioxidant compounds structurally related to phenols, such as aromatic amines, phenothiazines, pyridinols, pyrimidinols, and thiols [34].

The C/N ratio of humic materials is supposed to reflect a depletion of amino acid content along with a dominant accumulation of aromatic compounds with increasing HS age [2]. As a result, more transformed HS possess an aromatic backbone enriched with phenolic and quinonid units [87]. The calculated negative significant correlation r = −0.40 between C/N and the metoxyl carbon C_CH3O_ content (Table S1), related to unaltered lignin fragments in the HS structure, is in line with this idea. Thus, a positive correlation between the AOC of HS and the C/N ratio most likely indicates a possible increase in the AOC of humic materials in the process of aging. The content of methoxyl fragments, in their turn, also negatively correlated (r = −0.56) with the AOC of HS (Table S1). Of particular interest is a direct correlation between AOC values and the content of anomeric aliphatic C atoms C_OCO_ and О-substituted methine groups C_CHO_, and the total content of carbohydrates ΣC_Carb_. Over the past decade, a variety of natural polysaccharides from functional and medicinal foods have attracted great interest due to their antioxidant functions, such as free radical scavenging [41], including peroxyl radicals [39,88]. One proposed model for the effects of free radical scavenging of carbohydrates is to subtract their anomeric hydrogen by free radicals and combine it to form a neutral molecule; then the generated alkoxyl radicals promote the intramolecular hydrogen abstraction reaction, which triggers the spirocyclization reaction to terminate the reactions of radical chains [40]. 

Some HS samples did not obey the general trends shown in Figure 2. The most dramatic examples can be seen for the AOC–TP and AOC–C_OCO_ pair content in soil HS, where similar TP or C_OCO_ values were related to very different AOC values. To define the important properties of HS that contribute the most to their antioxidant activity, a forward stepwise multiple linear regression model was developed. The AOC value (Table 3) was the dependent variable, while the structural parameters (Table 2) were independent variables. Among the variables studied, only four were responsible for the antioxidant capability of HS (Table 5).

**Figure 3:**
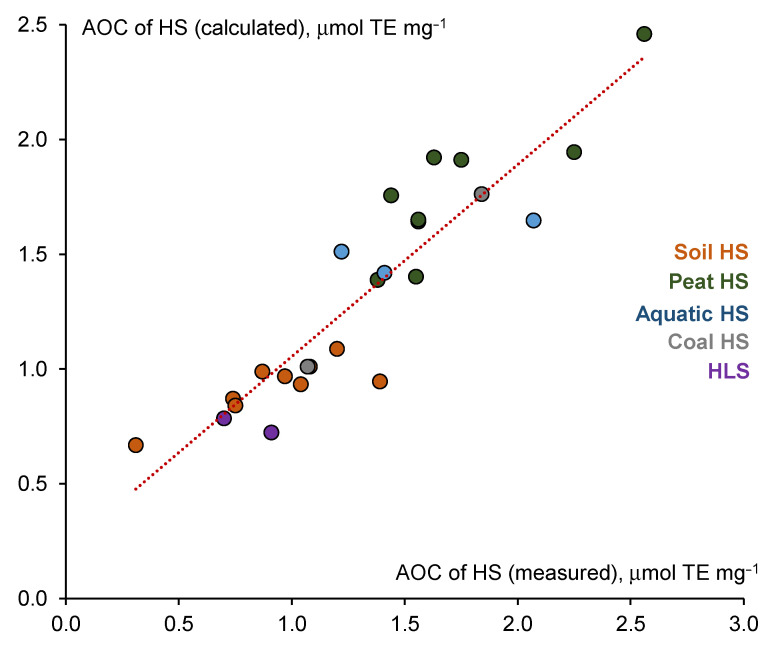
Relationship between measured and calculated AOC values of studied HS using a developed forward stepwise multiple linear regression model.

A high coefficient of multiple determination for the developed model is illustrated in Figure 3.

The four structural parameters contributing the most to the AOC values of HS were C/N, C_CHO_, C_CH3O_, and TP. An increase in C/N, C_CHO_, and TP led to an increase in AOC, whereas an increase in the C_CH3O_ content led to a decrease in AOC.

Free radical-scavenging antioxidants react with ROO^•^ peroxyl radicals by one of the following three reaction mechanisms [89]: hydrogen abstraction (hydrogen atom transfer (HAT)), radical adduct formation (RAF), or single electron transfer (SET). The relative importance of these reactions depends on the type of radicals, antioxidants, and microenvironment [35].

The ORAC method mostly involves the HAT reaction, where an antioxidant and a substrate (probe) compete for free radicals [81]. Thus, the observed direct correlation between AOC value and phenol content in HS aligns with the expected trends. Hydrogen atom transfer from phenol ArOH to peroxyl radical ROO^•^ is described as (2):

ArOH + ROO^•^ → ArO^•^ + ROOH (2)

A similar direct relationship between AOC value and phenol content in HS was reported by numerous studies [37,53,56,90,91]. The decisive role of phenols in quenching peroxyl radicals was demonstrated for other natural antioxidants as well [39,92,93].

Though phenols are known to play a key role as chain-breaking antioxidants, mainly via the HAT mechanism [34,35], quenching of the radical by the SET mechanism can also be expected. The predominant quenching of the radical by HAT or SET depends on phenol dissociation. For neutral forms of phenols (e.g., piceatannol, resveratrol), SET is much slower than HAT, which is not the case for phenolate ions, where the SET process becomes preferable [37,89]:

ArO^−^ + ROO^•^ → ArO^•^ + ROO^−^ (3)

A similar relationship is observed for several radicals where phenolate has much higher reactivity than their protonated forms [94]. Deprotonation of phenols is pronounced at pH > 7, at which point deprotonation of hydroxyl moieties generates semiquinone and quinone, facilitating electron transfer and boosting antioxidant activity [37,95]. Phenolate is 1000 or 1 million times more active than phenol because the H atom is heavier than the electron, and more difficult to transfer [96]. As a result, for anionic forms of phenols, the peroxyl radical scavenging activity takes place almost exclusively via the SET mechanism [89]. This phenomenon explains the observed negative value of the correlation coefficient between AOC and the carbon content of methoxyl group C_CH3O_ (Table S1). The methoxyl group increases the pKa of the phenol group to higher values, making the phenol group less acidic and thus decreasing the concentration of phenols in phenolate form at a certain pH value [97]. As the methoxyl group content in HS decreases along with an increase in C/N ratio due to microbial transformation [2,98], the opposite relationships of AOC versus C_CH3O_ and AOC versus C/N look rather logical.

The inverse relationship between AOC and the methoxyl group content at pH 7.4 obtained in this study is important, as methoxyl groups are usually reported to reduce the bond dissociation enthalpy (BDE) of the phenolic hydroxyl group and enhance the electron-donating ability of phenolic acids by reducing proton affinity and electron transfer enthalpy values. Altogether, these processes result in an increase in the antioxidant activity of methoxyl-substituted phenols, and an increase in methoxyl content brings about higher antioxidant activity of phenolic acids. At the same time, Chen and co-workers reported an increase in the antioxidant activity of phenolic acids along with an increase in methoxyl group content using a ferric ion reducing antioxidant power (FRAP) assay [99]. We believe that the observed inconsistency might result from a relatively high pH value used in the ORAC assay (pH 7.4) compared to other methods (pH 3.6 for FRAP assay).

Another important finding of this study is the observed significant contribution of carbohydrate moieties to the AOC value of HS against peroxyl radicals. Nominally, statistically significant direct correlations were obtained for AOC and the content of anomeric aliphatic C atoms (C_OCO_) and О-substituted methine groups (C_CHO_; Table S1). Moreover, C_CHO_ was selected as a predictive variable for AOC by forward stepwise regression (Table 5).

Although carbohydrates and polysaccharides represent an important and abundant class of free radical scavengers [6,31,34,66,67,68,69,70,100], these non-phenolic antioxidants are not considered to be strong antioxidants due to their low content of electron-donating aldehyde or ketone groups in the structure [52]. Besides, the observed antioxidant activity in this case is often related to impurities such as phenolic and/or protein components rather than to the carbohydrate moieties themselves [39,40,52].

However, Hernandez-Marin and Martínez, based on a theoretical study, concluded that direct scavenging of hydroxyl radicals OH^•^ was by carbohydrates C_m_(H_2_O)_n_ via the HAT mechanism [100]:

C_m_(H_2_O)_n_ + OH^•^ → C_m_HO(H_2_O)_n−1_^•^ + H_2_O (4)

There was no pronounced regioselectivity for the abstraction of the hydrogen atom. Therefore, HAT from both any C–H or O–H bond to the radical can be hypothesized. Direct scavenging of hydroperoxyl radical OOH^•^ by carbohydrates was considered less likely, since according to the modelling results, hydrogen atom abstraction was possible only from one position of a single studied carbohydrate (sucrose) [100]. Another proposed model is based on the observed increased antioxidant ability of carbohydrate polymers vs. monosaccharides. The authors speculated that the weak free radical scavenging activity of monosaccharides is due to the abstraction of anomeric hydrogen. The enhanced antioxidant activity of the polymers over the monomeric form may be due to the greater ease of abstraction of anomeric hydrogen from an internal glucose unit rather than from the reducing end [101]. However, further verification experiments to confirm the model have not been conducted [40]. Additionally, it is believed that the antioxidant activity of phenolic acids can be increased by modifying them with sugars. For instance, esterification of ferulic acid with arabinoxylan resulted in feruloyl arabinoxylo-oligosaccharide with much stronger antioxidant activity compared to free ferulic acid. The activity increased along with an increased number of sugar moieties; the glycosyl group by itself showed no activity [102].

Regardless of the mechanism determining the antioxidant activity of sugars, the results obtained in this study clearly demonstrate the dependence of the AOC of HS on the content of carbohydrate fragments in their structure.

**Figure 4:**
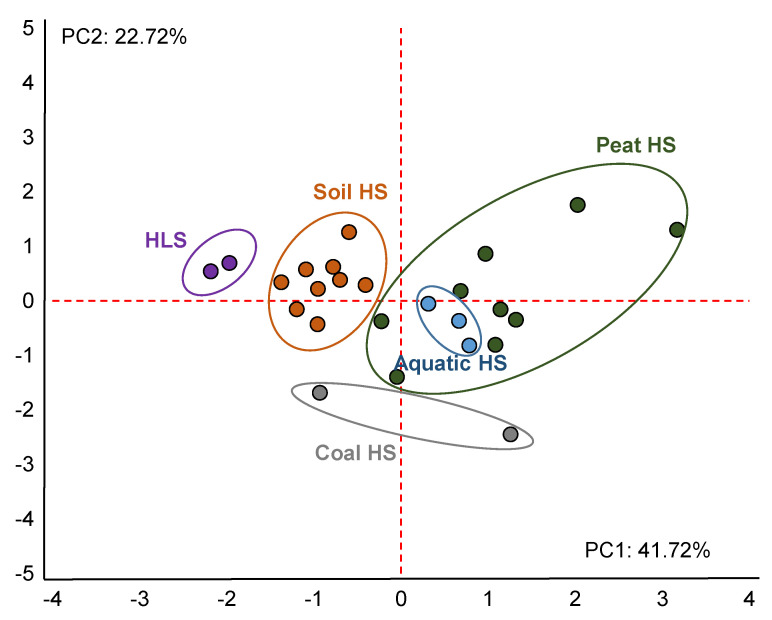
Projection of cases on a factor plane: PCA result for classification of HS used in this study based on their physical–chemical properties, selected after forward stepwise regression (C/N, content of C_CHO_, C_CH3O_, TP).

The PCA performed for the selected structural parameters of HS to determine their antioxidant capability against peroxyl radicals resulted in a separation of all samples by the first two factors (Figure 4).

Figure 4 shows the PCA results, with 64.44% of the total variance explained by the properties determining the AOC of HS against hydroxyl radicals. The samples from the different natural sources built distinct clusters, except for aquatic HS. These HS were isolated from HLS, soil HS, and coal HS, but formed a group with peat HS. The latter most likely resulted from the fact that the Suwannee River, which was a source for the aquatic samples in this work, is a blackwater river rising in the Okefenokee Swamp [103]. The HLS were clustered and different from coal HS and aquatic HS in PC1 (41.72%) because of the high content of methoxyl carbon C_CH3O_ derived from lignin. Aquatic and coal HS were clustered with negative values because of the high C/N values. Soil HS were clustered from peat HS in PC2 (22.72%) due to the lower content of О-substituted methine groups C_CHO_.

So, based on a statistical analysis of the obtained data, we believe that, apart from phenol fragments, the presence of non-phenolic moieties such as carbohydrates impart antioxidant activity to HS against peroxyl radicals. To verify this hypothesis, more experiments are required to assess the antioxidant activity of HS using biological models.

## 5. Conclusions

This is the first systematic study conducted on a broad set of humic materials of different origins and fractional compositions with respect to the quenching capability of HS against biologically relevant peroxyl radicals with the ORAC method. The findings demonstrate that humic materials derived from different environments possess distinct antioxidant activity against peroxyl radicals. The high measured AOC values reached those for ascorbic acid and vitamin E. They were observed for the most active representatives: peat fulvic acids enriched with both phenolic and sugar moieties. Four structural parameters of HS were identified as major contributors to antioxidant capacity: total phenol content, atomic C/N ratio, and content of O-substituted methine and methoxyl groups. The results obtained clearly demonstrate that the antioxidant activity of HS against peroxyl radicals depends on both phenolic and non-phenolic moieties in their structure. For the first time, the substantial contribution of sugar moieties to the AOC of humic materials is revealed. The possible mechanisms of these phenomena are discussed. A conclusion is made regarding the complex character of AOC dependence on HS structure due to the very complex molecular organization of these natural systems, consisting of both high- and low-molecular-weight compounds. Fine fractionation of HS beyond traditional separation into humic and fulvic acids is highly recommended in order to determine more consistent relationships between structure and antioxidant properties. Deeper insight into the molecular composition of HS using high-resolution Fourier-transform ion cyclotron mass spectrometry might also advance our understanding of the hidden relationships between structure and properties for such chemically complex molecular systems.

## Figures and Tables

**Table 1:** Humic substances used in the study and their sources.

HS Index	HA Type	Source
Soil		
SHA-PG96	HA	Albic Retisol
SHA-PW96	HA	Albic Retisol
SHA-PG94	HA	Albic Retisol
SHA-CM94	HA	Gleyic Chernozem
SHA-PW94	HA	Albic Retisol
SHA-CTV94	HA	Endocalcic Chernozem
SFA-PP96	FA	Albic Retisol
SFA-PW96	FA	Albic Retisol
SFA-PG96	FA	Albic Retisol
Peat		
PHA-T398	HA	Lowland peat, sedge type
PHA-T798	HA	Lowland peat, woody type
PHA-T698	HA	Highland peat, sedge type
PFA-T398	FA	Lowland peat, sedge type
PFA-T698	FA	Highland peat, sedge type
PFA-T798	FA	Lowland peat, woody type
PHF-T398	HA+FA	Lowland peat, sedge type
PHF-T698	HA+FA	Highland peat, sedge type
PHF-T798	HA+FA	Lowland peat, woody type
Natural water		
SRHA	HA	Suwannee River (IHSS standard)
SRFA	FA	Suwannee River (IHSS standard)
SRDOM	DOM	Suwannee River (IHSS standard)
Coal		
СHA-AGK	HA	Biotechnology Ltd. (RF)
CHA-ALD	HA	Aldrich (Germany)
Humic-like substances		
HLS-45	HA	Oat straw solubilized by *Trametes maxima* 0275
HLS-70	HA	Oat straw solubilized by *Trametes maxima* 0275

**Table 2:** Structural characteristics of humic materials used in this study.

HS Index	Atomic Ratio ^1^			Content of Carbon in Structural Fragments Determined by ^13^CNMR Spectroscopy as Integral Intensity (%) ^2^														TP ^3^, μmol TE mg^−1^
	O/C	H/C	C/N	C_C=O_	C_COO_	С_ArO_	С_Ar_	C_OCO_	C_CHO_	C_CH2O_	C_CH3O_	C_CHn_	∑С_Ar_	∑C_Сarb_	∑C_Alk_	∑C_Alk-O_	∑C_Ar_/∑C_Alk_	
	Soil																	
SHA-PG96	0.48	0.93	15.9	2.0	17.0	11.0	31.0	2.0	10.0	2.0	4.0	22.0	42.0	14.0	40.0	18.0	1.05	1.378 ± 0.001
SHA-PW96	0.46	1.10	14.0	4.0	19.0	9.0	25.0	2.0	8.0	2.0	4.0	26.0	34.0	12.0	42.0	16.0	0.81	1.230 ± 0.005
SHA-PG94	0.45	1.00	13.6	1.1	15.2	12.8	32.8	3.0	10.0	2.1	5.8	17.5	45.6	15.1	38.4	20.9	1.19	1.392 ± 0.001
SHA-CM94	0.34	0.66	13.8	1.5	14.3	8.7	48.1	2.0	6.0	0.8	4.6	14.2	56.8	8.8	27.6	13.4	2.06	1.760 ± 0.001
SHA-PW94	0.46	0.92	11.5	1.0	17.0	13.0	33.0	2.0	8.0	2.5	5.5	17.0	46.0	12.5	35.0	18.0	1.31	1.753 ± 0.001
SHA-CTV94	0.42	0.62	15.9	3.0	15.0	11.0	43.0	3.0	7.0	2.0	3.0	13.0	54.0	12.0	28.0	15.0	1.93	0.510 ± 0.001
SFA-PP96	0.54	0.91	11.3	2.0	23.0	7.0	19.0	3.0	15.0	3.0	4.0	22.0	26.0	21.0	47.0	25.0	0.55	1.167 ± 0.001
SFA-PW96	0.58	0.94	17.2	4.0	19.5	9.8	23.5	2.0	10.0	2.5	3.5	25.1	33.3	14.5	43.1	18.0	0.77	1.523 ± 0.001
SFA-PG96	0.61	0.88	19.4	3.1	18.0	13.1	28.2	2.0	11.0	2.4	4.2	17.6	41.3	15.4	37.2	19.6	1.11	1.176 ± 0.001
	Peat																	
PHA-T398	0.44	0.87	25.9	8.7	15.5	7.4	32.4	7.4	4.4	2.0	4.1	18.2	39.7	13.8	36.1	17.9	1.10	2.743 ± 0.001
PHA-T798	0.49	0.87	46.2	4.1	11.0	9.4	31.7	3.7	14.2	2.7	3.4	19.8	41.1	20.6	43.8	24.0	0.94	2.986 ± 0.001
PHA-T698	0.55	0.91	37.9	1.7	8.6	7.2	35.4	2.6	12.3	2.7	4.0	25.7	42.5	17.6	47.3	21.6	0.90	3.353 ± 0.001
PFA-T398	0.66	0.76	120	3.1	17.0	9.0	28.1	4.9	11.8	2.3	3.8	20.0	37.1	19.0	42.8	22.8	0.87	1.239 ± 0.001
PFA-T698	0.51	1.03	101	1.6	10.9	6.9	24.4	6.9	26.1	3.1	1.8	18.3	31.3	36.1	56.2	37.9	0.56	2.368 ± 0.001
PFA-T798	0.60	1.00	74.1	2.2	11.8	10.9	24.0	9.6	24.1	3.5	2	11.9	34.9	37.2	51.1	39.2	0.68	1.602 ± 0.001
PHF-T398	0.49	1.10	35.5	3.4	10.6	9.8	32.9	6.7	11.0	3.0	5.6	16.9	42.7	20.7	43.2	26.3	0.99	2.398 ± 0.001
PHF-T698	0.54	0.91	48.5	3.7	10.9	8.4	24.4	7.7	19.8	4.8	4.2	16.2	32.8	32.3	52.7	36.5	0.62	2.275 ± 0.001
PHF-T798	048	0.87	53.0	3.5	10.4	8.7	28.1	8.8	15.2	3.5	4.4	17.4	36.8	27.5	49.3	31.9	0.75	2.275 ± 0.001
	Natural water																	
SRHA	0.60	0.97	52.5	8.5	18.1	10.7	26.6	6.3	8.9	1.9	3.4	15.6	37.3	17.1	36.1	20.5	1.03	2.504 ± 0.001
SRFA	0.62	0.99	91.1	7.4	19.6	10.9	22.3	4.4	10.0	1.8	4.0	19.6	33.2	16.2	39.8	20.2	0.83	1.537 ± 0.001
SRDOM	0.61	0.95	55.6	6.9	19.9	8.0	23.7	5.2	11.3	2.0	4.0	19.0	31.8	18.5	41.5	22.5	0.76	1.791 ± 0.001
	Coal																	
СHA-AGK	0.32	0.79	112	0.5	16.9	10.0	47.8	0	0	1.0	3.0	20.8	57.8	1.0	24.8	4.0	2.33	2.348 ± 0.001
CHA-ALD	0.31	0.81	77.0	1.0	15.0	13.0	43.0	0	0	1.0	6.0	21.0	56.0	1.0	28.0	7.0	2.00	1.521 ± 0.001
	Humic-like substances																	
HLS-45	0.37	1.22	16.1	3.8	10.9	11.2	25.6	6.8	13.7	4.4	8.4	15.1	36.8	24.9	48.4	33.3	0.76	1.481 ± 0.003
HLS-70	0.55	1.03	23.7	4.0	15.1	10.9	26.8	4.8	12.7	4.2	9.0	12.5	37.6	21.7	43.2	30.7	0.87	1.381 ± 0.001

^1^ H/C, O/C and N/C ratios were calculated on an ash- and water-free basis. ^2^ Content of carbon in structural fragments was determined by ^13^C NMR spectroscopy as integral intensity (%) of the following spectral regions (ppm): 220–189 (C_C=O_), 189–168 (C_COO_), 168–145 (C_Ar-O_), 145–108 (C_Ar_), 108–91 (C_OCHO_), 91–66 (C_CHO_), 66–59 (C_CH2O_), 59–48 (C_CH3O_), 48–0 (C_CHn_). ^3^ Values are means ± SD (n = 3).

**Table 3:** AOC values of HS samples from different environments used in this study as measured by the ORAC method.

HS Index	AOC, μmol TE mg^−1^
Soil	
SHA-PG96	0.87 ± 0.08 ^bc^
SHA-PW96	0.74 ± 0.04 ^bc^
SHA-PG94	0.75 ± 0.03 ^bc^
SHA-CM94	0.97 ± 0.05 ^d^
SHA-PW94	1.04 ± 0.05 ^de^
SHA-CTV94	0.31 ± 0.02 ^a^
SFA-PP96	1.08 ± 0.06 ^def^
SFA-PW96	1.20 ± 0.06 ^efg^
SFA-PG96	1.39 ± 0.06 ^hi^
Peat	
PHA-T398	1.55 ± 0.03 ^hij^
PHA-T798	1.63 ± 0.05 ^jk^
PHA-T698	1.75 ± 0.03 ^kl^
PFA-T398	1.56 ± 0.08 ^hij^
PFA-T698	2.56 ± 0.06 ^m^
PFA-T798	2.25 ± 0.13 ^m^
PHF-T398	1.38 ± 0.03 ^gh^
PHF-T698	1.44 ± 0.05 ^hi^
PHF-T798	1.56 ± 0.08 ^ij^
Natural water	
SRHA	2.07 ± 0.09 ^m^
SRFA	1.22 ± 0.04 ^fg^
SRDOM	1.41 ± 0.05 ^hi^
Coal	
СHA-AGK	1.84 ± 0.07 ^l^
CHA-ALD	1.07 ± 0.06 ^def^
Humic-like substances	
HLS-45	0.70 ± 0.05 ^b^
HLS-70	0.91 ± 0.08 ^cd^

Values are means ± SD (n = 4). Values denoted with different letters within a column are significantly different at *p* < 0.05 according to Tukey’s HSD test.

**Table 4:** AOC of individual antioxidants estimated by the ORAC method.

Compound	AOC, μmol TE mg^−1^
Ascorbic acid	2.32 ± 0.21
Vitamin E	2.95 ± 0.16
Gallic acid	6.35 ± 0.25
Syringic acid	7.67 ± 0.39
Hydroxybenzoic acid	13.11 ± 0.63
Sinapic acid	15.71 ± 1.01
Vanillic acid	20.46 ± 1.14
Ferulic acid	23.64 ± 1.18
Coumaric acid	30.58 ± 1.32

Values are means ± SD (n = 4).

**Table 5:** Parameters of a forward stepwise multiple linear regression model.

Parameter	Value	−95%	+95%
Intercept	0.432	−0.087	0.952
C/N	0.006	0.003	0.009
C_CHO_, %	0.026	0.009	0.042
C_CH3O_, %	−0.077	−0.143	−0.010
TP, μmol mg^−1^	0.369	0.216	0.522
Multiple determination coefficient	0.91		
Adjusted multiple determination coefficient	0.80		

## Supplementary Materials

The following are available online at https://www.mdpi.com/article/10.3390/polym13193262/s1: Table S1: Pearson correlation coefficients between physical–chemical HS properties and their AOC, Table S2: Homogeneous groups of HS used in the study according Tukey’s HSD test; variable AOC, μmol TE mg^−1^; *p* < 0.05.
