# Supplementary material for: A Systematic Study of the Antioxidant Capacity of Humic Substances against Peroxyl Radicals: Relation to Structure
Source: Polymers (Basel). 2021 Sep 25;13(19):3262. doi: 10.3390/polym13193262 (PMC8512611; doi:10.3390/polym13193262)
Supplement: Supplementary file 1 [file polymers-13-03262-s001.zip › polymers-1382793-supplementary.pdf]

Supplementary Materials

A Systematic Study of the Antioxidant Capacity of Humic Substances against Peroxyl Radicals: Relation to Structure

Olga I. Klein [1], Natalia A. Kulikova [1,2,*], Andrey I. Konstantinov [3], Maria V. Zykova [4] and Irina V. Perminova [3]

Table S1. Pearson correlation coefficients between physical-chemical HS properties and their AOC [1].

	**AOC**	**O/C**	**H/C**	**C/N**	**$C_{C=O}$**	**C_{COO}**	**C_{ArO}**	**C_{Ar}**	**C_{OCO}**	**C_{CHO}**	**C_{CH2O}**	**C_{CH3O}**	**C_{CHn}**	**ΣC_{Ar}**	**ΣC_{Carb}**	**ΣC_{Alk}**	**ΣC_{Alk-O}**	**$\Sigma C_{Ar}/\Sigma C_{Alk}$**	**TP**
AOC	1.00																		
O/C	0.36	1.00																	
H/C	0.08	0.21	1.00																
C/N	**0.65**	0.18	-0.14	1.00															
$C_{C=O}$	0.11	**0.42**	0.21	0.00	1.00														
C_{COO}	-0.29	0.23	-0.11	-0.11	0.24	1.00													
C_{ArO}	-0.39	-0.17	0.05	-0.16	-0.21	0.13	1.00												
C_{Ar}	-0.19	**-0.73**	**-0.64**	0.03	**-0.42**	-0.22	0.22	1.00											
C_{OCO}	**0.44**	0.37	0.37	0.17	**0.43**	**-0.45**	-0.33	**-0.51**	1.00										
C_{CHO}	**0.46**	**0.52**	**0.40**	0.13	-0.04	-0.39	-0.34	**-0.65**	**0.68**	1.00									
C_{CH2O}	0.10	0.32	**0.53**	-0.14	0.03	**-0.45**	-0.15	**-0.60**	**0.65**	**0.74**	1.00								
C_{CH3O}	**-0.56**	-0.27	0.37	**-0.40**	-0.03	-0.10	0.38	0.02	-0.06	-0.23	0.31	1.00							
C_{CHn}	0.00	0.05	0.06	0.04	-0.08	0.30	-0.29	-0.10	**-0.50**	-0.24	-0.31	-0.24	1.00						
ΣC_{Ar}	-0.27	**-0.72**	**-0.58**	-0.01	**-0.44**	-0.18	**0.43**	**0.98**	**-0.54**	**-0.68**	-0.59	0.11	-0.16	1.00					
ΣC_{Carb}	**0.46**	**0.50**	**0.44**	0.12	0.10	**-0.45**	-0.35	**-0.67**	**0.83**	**0.97**	**0.81**	-0.14	-0.35	**-0.69**	1.00				
ΣC_{Alk}	0.39	**0.52**	**0.59**	0.07	0.07	-0.38	**-0.44**	**-0.77**	0.66	**0.90**	**0.81**	-0.06	0.04	**-0.82**	**0.91**	1.00			
ΣC_{Alk-O}	0.36	**0.45**	**0.52**	0.05	0.10	**-0.47**	-0.28	**-0.67**	**0.83**	**0.93**	**0.87**	0.05	**-0.40**	**-0.68**	**0.98**	**0.90**	1.00		
$\Sigma C_{Ar}/\Sigma C_{Alk}$	-0.27	**-0.71**	**-0.64**	0.05	-0.35	0.06	0.37	**0.94**	**-0.61**	**-0.76**	**-0.70**	0.02	-0.13	**0.95**	**-0.78**	**-0.91**	**-0.78**	1.00	
TP	**0.64**	-0.02	0.10	0.23	0.19	**-0.54**	**-0.46**	0.09	0.27	0.12	0.08	-0.19	0.17	-0.02	0.07	0.23	0.14	-0.12	1.00

[1] The values in bold denote statistically significant correlation coefficients at $p < 0.05$.
H/C, O/C and N/C ratios are calculated on ash- and water-free basis.
Content of carbon in the structural fragments is determined by ^{13}C NMR spectroscopy as the integral intensity (%) of the following spectral regions (ppm): 220–189 ($C_{C=O}$), 189-168 (C_{COO}), 168-145 (C_{Ar-O}), 145-108 (C_{Ar}), 108–91 (C_{OCHO}), 91–66 (C_{CHO}), 66–59 (C_{CH2O}), 59–48 (C_{CH3O}), 48–0 (C_{CHn}).
TP is measured in μmol TE mg^{-1}.

Table S2. Homogenous groups of HS used in the study according the Tukey HSD test; variable AOC, μmol TE mg^{-1}; $p<0.05$.

HS index	a	b	c	d	e	f	g	h	i	j	k	l	m	n
SHA-CTV94	****													
HLS-45		****												
SHA-PW96		****	****											
SHA-PG94		****	****											
HLS-70			****	****										
SHA-CM94				****										
SHA-PW94				****	****									
CHA-ALD				****	****	****								
SFA-PP96				****	****	****								
SFA-PW96					****	****	****							
SRFA						****	****							
PHF-T398							****	****						
SFA-PG96								****	****					
SRDOM								****	****					
PHF-T698								****	****					
PFA-T398								****	****	****				
PHA-T398								****	****	****				
PHF-T798									****	****				
PHA-T798										****	****			
PHA-T698											****	****		
CHA-AKG												****		
SRHA													****	
PFA-T798													****	
PFA-T698														****
